# Consensus Report on *Shigella* Controlled Human Infection Model: Introduction and Overview

**DOI:** 10.1093/cid/ciz886

**Published:** 2019-12-09

**Authors:** Calman A MacLennan, Anastazia Older Aguilar, A Duncan Steele

**Affiliations:** 1 Bill & Melinda Gates Foundation, London, United Kingdom; 2 Bill & Melinda Gates Foundation, Seattle, Washington

**Keywords:** *Shigella*, controlled human infection model, human infection studies

## Abstract

In recent years, controlled human infection models (CHIMs) have become available for a range of infectious agents and have proved invaluable for understanding the disease process, pathogenesis, and mechanisms of immunity. CHIM studies have also contributed significantly to advancing development of a number of vaccines by providing an indication of vaccine efficacy. The *Shigella* CHIM has been established in 3 sites in the United States, and it is likely that the CHIM will play an important regulatory role for advancing the range of *Shigella* vaccine candidates that are currently in development. This supplement describes the harmonization of best practices across sites, with a view to maximizing the contribution that CHIM studies can make to *Shigella* vaccine development.

Controlled human infection models (CHIMs) involve the deliberate administration of a predetermined quantity of infectious agent to healthy human volunteers. Such an infectious challenge is followed by carefully monitoring the clinical course of volunteers, using both clinical observation and laboratory investigation, through to a predefined clinical endpoint, often followed by the administration of antibiotics. Subsequent monitoring takes place until complete clearance of the infection can be proven with reasonable confidence. Safety is paramount among such studies, along with ensuring that shedding and transmission to the environment do not occur. Prior to enrollment, volunteers are carefully screened against predefined inclusion and exclusion criteria, to minimize the risk of a serious adverse event occurring. While taking part in a CHIM study, volunteers are subjected to continuous monitoring by clinical staff, often in a residential clinical facility.

CHIMs have been established for a range of pathogens including bacteria (*Vibrio cholerae* [1], *Salmonella enterica* serovar Typhi [2], and *Shigella flexneri* 2a and *Shigella sonnei* [3]), viruses (influenza [4]), and parasites (*Plasmodium falciparum* [5]). Where deliberate infection with a pathogen is not possible on safety grounds (eg, tuberculosis), live attenuated vaccines (eg, BCG [6]) have been used as a surrogate for the pathogen.

CHIM studies permit the detailed analysis of the infectious disease process. They provide valuable opportunities to make key insights into infectious disease pathogenesis and mechanisms of immunity to infection, including the identification of correlates of protection. Importantly for this supplement, such studies are proving increasingly valuable in the clinical development of candidate vaccines. CHIM studies permit an early understanding of the efficacy of candidate vaccines under development. This allows down-selection prior to making the large financial commitment required to take a candidate vaccine through late-stage clinical development.

More recently, CHIM studies themselves have proven pivotal in late-stage clinical development: first, for the licensing of the Vaxchora cholera vaccine (PaxVax, Inc), where a field efficacy study was not possible [7, 8]. Second, and more recently, a CHIM study provided supportive evidence for the World Health Organization (WHO) prequalification of a first typhoid conjugate vaccine, Typbar TCV (Bharat Biotech, Hyderabad, India) [2]. The vaccine was licensed in India several years previously based on immunogenicity data assessed against field efficacy of a historic typhoid conjugate vaccine in Vietnam [9]. The clinical protection observed in the CHIM study contributed to a strong policy recommendation from the WHO Strategic Advisory Group of Experts on Immunization [10].

Shigellae are gram-negative bacteria that cause both acute diarrheal disease and dysentery in young children and in adults [11]. Our understanding of the magnitude of the global burden of disease attributable to *Shigella* has been greatly enhanced over the past few years through large epidemiological studies. Reanalysis of data from the Global Enteric Multicenter Study in low- and middle-income countries (LMICs) [12] using molecular diagnostics indicates that *Shigella* is the most attributable cause of moderate to severe diarrhea in children <5 years of age and is particularly prevalent among children aged 1–5 years [13]. It is now apparent that shigellosis is associated with growth stunting among children in LMICs [14]. Moreover, *Shigella* is on the WHO pathogen priority list due to growing levels of antimicrobial resistance among field isolates, particularly to fluoroquinolones [15].

There is a long history of CHIM studies involving *Shigella*. Following initial attempts in the 1940s [16], the first informative studies were conducted by the team of Drs Herbert DuPont and Samuel Formal at the University of Maryland, first with *S. flexneri* 2a in the 1960s [17] and subsequently with *S. sonnei* [18]. The *S. sonnei* CHIM was also established at the US Armed Forces Research Institute of Medical Sciences facility in Bangkok to provide a CHIM in an endemic setting [19]. When subsequently used to test for efficacy with the live attenuated *S. sonnei* vaccine WRSS1, the CHIM identified no efficacy. This finding could be due in part to the low attack rate of dysentery at 20% (compared with 75% in a previous study in Thailand) and the small numbers of subjects investigated (10 vaccinees and 10 controls) [20]. In addition, the CHIM study had no clear primary clinical endpoint, thereby emphasizing the need for standardization of the model across sites.

There have been several issues identified with the potential utilization of the *Shigella* CHIM, which have been reviewed previously [3] and include the variety of *Shigella* strains that might be required and the dose ranging of each of these strains; standardized challenge strains with expected diarrhea attack rates; the administration with or without buffer and specified buffering solutions; clinical endpoints for standardized evaluation across models; and clinical sampling and assay standardization. To fully optimize the potential use of the *Shigella* CHIM for advancing multiple vaccine candidates, these issues need to be addressed. This was the topic of an earlier workshop that served to bring together some of the key stakeholders to assess these key questions [21]. The manuscripts reported in this supplement are the next step to achieving an optimal pathway for utilizing the CHIM model to advance effectively *Shigella* vaccine candidates.

The *Shigella* CHIM has been established/reestablished at 3 sites in the eastern United States: in Baltimore, both at Johns Hopkins University and the University of Maryland, and in Cincinnati, at the Cincinnati Children’s Hospital Medical Center. With multiple *Shigella* candidate vaccines in clinical development, and several of these being tested in clinical trials involving CHIM, it is important to achieve harmonization of the model, including the model itself, clinical endpoints, and immunological assays across these 3 sites, as mentioned above.

Harmonization is needed to ensure sharing of best practices and to permit comparability of *Shigella* CHIM study results across sites. Among other studies, recently a monovalent bioconjugate *S. flexneri* 2a vaccine (Limmatech, GlaxoSmithKline [GSK]) has been tested in a CHIM study at Johns Hopkins University [22]. A *S. sonnei* monovalent outer membrane vesicle–based vaccine (GSK Vaccines Institute for Global Health) is currently being tested in Cincinnati [23]. Plans are advanced for a monovalent synthetic O-antigen–based conjugate vaccine (Institut Pasteur) to be tested at the University of Maryland. There are other vaccine constructs that have been, or will need to be, evaluated in CHIM studies, including live attenuated strains, killed whole-cell candidates, and other subunit vaccines [24].

With the objective of harmonizing the *Shigella* CHIM across sites, the Bill & Melinda Gates Foundation convened a set of workshops in 2017 and 2018 bringing together global experts on CHIM and *Shigella*, including representatives from the 3 *Shigella* CHIM sites in the United States, vaccine developers, and global health policy makers. These workshops resulted in the establishment of a consensus position across the field, the outputs of which are contained in this supplement to *Clinical Infectious Diseases*. The supplement consists of 3 articles, in addition to this introduction, focusing on the general conduct of the *Shigella* CHIM (Talaat et al), *Shigella* CHIM clinical endpoints (MacLennan et al), and *Shigella* CHIM laboratory assays (Kaminski et al).

Our intention is that the reports will form the guidelines for conducting *Shigella* CHIM studies going forward. Feedback on the degree of success of the guidelines will provide the best indication of their suitability and will be important for iterative improvement in the future as more *Shigella* CHIM study data become available. Finally, we hope that this process of harmonization of the CHIM in the *Shigella* field will form a template for a similar process to take place in other infectious disease areas, particularly where CHIMs have been established at multiple sites.

## Notes

***Supplement sponsorship.*** This supplement is sponsored by the Bill & Melinda Gates Foundation.

***Potential conflicts of interest.*** The authors report no potential conflicts of interest. All authors have submitted the ICMJE Form for Disclosure of Potential Conflicts of Interest. Conflicts that the editors consider relevant to the content of the manuscript have been disclosed.
